# A comprehensive comparative assessment of eight risk stratification systems for thyroid nodules in the elderly population

**DOI:** 10.3389/fonc.2023.1265973

**Published:** 2023-11-15

**Authors:** Xiao Ma, Jing Yu, Yuanjing Huang, Yiyang Cui, Kefei Cui

**Affiliations:** Department of Ultrasound, The First Affiliated Hospital of Zhengzhou University, Zhengzhou, China

**Keywords:** thyroid nodules, risk stratification systems, elderly, ultrasonography, Thyroid Imaging Reporting and Data System

## Abstract

**Objective:**

This study aims to investigate the diagnostic value of eight risk stratification systems (RSSs) for thyroid nodules in the elderly and explore the reasons in comparison with a younger group.

**Methods:**

Cases of thyroid nodules that underwent ultrasound examination with thyroidectomy or fine-needle aspiration (FNA) at our hospital between August 2013 and March 2023 were collected. The patients were categorized into two groups: an elderly group (aged ≥60) and a younger group (aged <60). Eight RSSs were applied to evaluate these nodules respectively.

**Results:**

The malignant rate in the elderly group was significantly lower than that in the younger group (28.2% vs. 49.6%, P=0.000). There were statistically significant differences in nodule diameter, multiplicity, composition, echogenicity, orientation, margin, and echogenic foci between the elderly and younger groups (P<0.05). Among the eight RSSs evaluated in elderly adults, the artificial intelligence-based Thyroid Imaging Reporting and Data System (AI TIRADS) demonstrated the highest overall diagnostic efficacy, but with relatively high unnecessary FNA rate (UFR) and missed cancer rate (MCR) of 55.0% and 51.3%, respectively. By modifying the size thresholds, the new AI TI-RADS achieved the lowest UFR and MCR while maintaining nearly the lowest FNA rate (FNAR) among all the RSSs (P=0.172, 0.162, compared to the ACR and original AI, respectively, but P<0.05 compared to the other six RSSs).

**Conclusion:**

Among the eight RSS systems, AI demonstrated higher diagnostic efficacy in the elderly population. However, the size thresholds for FNA needed to be adjusted.

## 1 Introduction

With the widespread application of imaging techniques, the prevalence of thyroid nodules in adults reaches approximately 19%–68%, and it tends to increase with age (1, 2). To aid clinicians in determining suitable management strategies for the growing number of thyroid nodules, various versions of ultrasound (US)-based risk stratification systems (RSSs) have been developed in recent years. The commonly utilized RSSs can be broadly classified into two groups: the “point-based” system and the “pattern-based” system. The point-based system comprises the Thyroid Imaging Reporting and Data System (TIRADS) established by Kwak et al. (Kwak) (3), American College of Radiology (ACR) (4), Benjamin et al. with an artificial intelligence algorithm (AI) (5), and the Chinese (C-TIRADS) (6). The pattern-based system comprises the American Thyroid Association (ATA) guideline (2), the American Association of Clinical Endocrinologists, American College of Endocrinology, and Associazione Medici Endocrinology (AACE/ACE/AME) guideline (7), European Thyroid Association (EU) TIRADS (8) and Korean Society of Thyroid Radiology (K-TIRADS) (9). All these systems have exhibited excellent diagnostic performance (10–14). However, studies showed that age is a confounding factor that cannot be overlooked (15, 16).

Age is associated with an increased incidence of thyroid nodules, a lower malignancy rate, and a higher proportion of invasive nodules (17). This implies that RSSs designed for the general population may not necessarily be applicable to older patients. To the best of our knowledge, there is currently no comparative study of these eight systems specifically focusing on thyroid nodules in the elderly population. This study aims to analyze thyroid nodules in elderly patients using the eight RSS systems, investigate the optimal diagnostic system, and explore whether the established biopsy thresholds are applicable to older individuals.

## 2 Materials and methods

### 2.1 Patients

**Figure 1:**
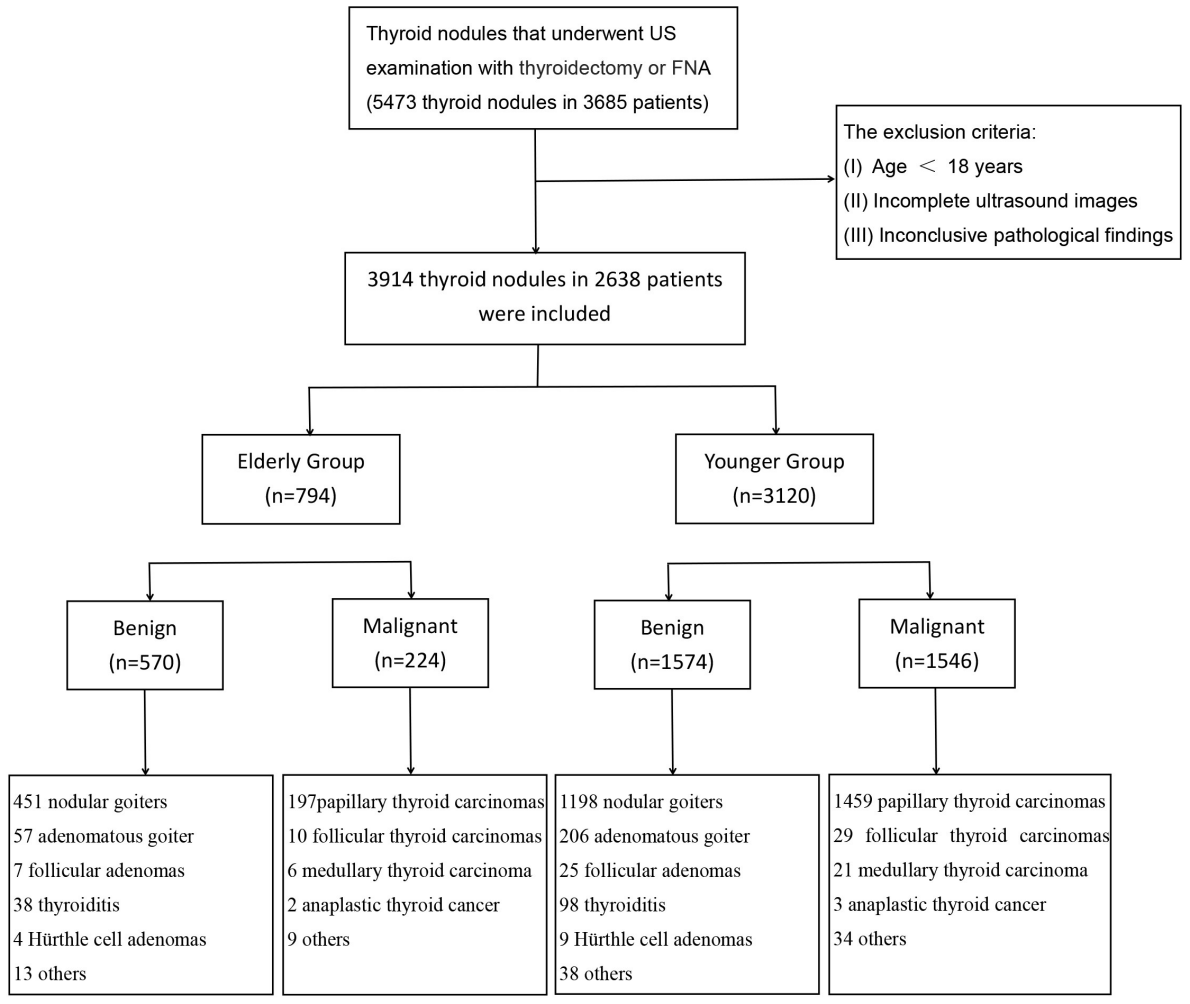
Flowchart of study subject inclusion. US, ultrasound; FNA, fine needle aspiration; n, number.

The Scientific Research and Clinical Trials Ethics Committee of the First Affiliated Hospital of Zhengzhou University of China granted approval for this retrospective study and waived the requirement for written informed consent for data usages. The study was conducted from August 2013 to March 2023 on a cohort of 5473 thyroid nodules in 3685 patients who received thyroid US exams and thyroid surgery or fine-needle aspiration (FNA) at our hospital. A total of 3914 thyroid nodules in 2638 patients were included in this study after meeting the exclusion criteria. Then, the nodules were divided into two groups according to the ages: elderly group (≥60 years old, 794 nodules in 504 patients) and younger group (<60 years old, 3120 nodules in 2134 patients) (**Figure 1**). The definition of 60 years as the age standard was based on our country’s regulations, medical situation, and previous literature (18). The exclusion criteria were as follows: (I) Age < 18 years. (II) Incomplete ultrasound images. (III) Inconclusive pathological results. If surgery had been performed, then the postoperative pathology resulted prevail. If no surgery was done, the results of the FNA was applied. Cytology was classified according to the Bethesda System (19). Bethesda V and VI were considered malignant, Bethesda II were considered benign. Bethesda classes I, III or IV were excluded as uncertain outcomes. In the elderly group, 456 nodules were confirmed by postoperative pathology, consisting of 271 benign and 185 malignant cases. Additionally, 338 nodules were confirmed through FNA, with 299 benign and 39 malignant nodules. In the younger group, there were 2011 nodules with pathological confirmation, comprising 713 benign and 1298 malignant cases. Among these, 1109 nodules were confirmed through FNA, including 861 benign and 248 malignant cases.

### 2.2 Thyroid ultrasound examination and image interpretation

One of two US specialists with 33 or 11 years of expertise in thyroid US did each examination with Aplio 300 or 500 (Toshiba Corporation, Tokyo, Japan) equipped with a 5-12 MHz linear array transducer. Two superficial sonographers (with 8 and 12 years of expertise analyzing thyroid US images), blinded to the biopsy results and the final pathological diagnoses were hired to assess the ultrasonic features of the nodules and classify them according to the ATA guidelines, ACR, AI, Kwak, EU, AACE/ACE/AME, C and K-TIRADS. US features included the size (the maximal diameter on US), composition (solid or almost solid, mixed cystic and solid, cystic, spongiform), echogenicity (hyperechoic, isoechoic, hypoechoic, markedly hypoechoic, anechoic), orientation (taller-than-wide, wider-than-tall), margin (smooth, ill-defined, irregular or lobulated, extrathyroidal extension) and echogenic foci (punctuate echogenic foci, peripheral calcifications, macrocalcification, comet-tail artifacts). It is worth noting that comet-tail artifacts were recorded only in the absence of microcalcification. Other types of calcifications could be selected simultaneously. When the two doctors had differing opinions, a third expert with 33 years of thyroid imaging experience participated in a joint discussion to reach a final decision. Before assessing the ultrasonic features, an interactive case-based training session was conducted using 30 representative thyroid nodules not included in this study. Then, FNA were determined based on the size thresholds of each guideline. It was worth noting that the unclassified nodules in the ATA guidelines were grouped with intermediate-suspicion categories, as in previous reports (20–23).

### 2.3 Statistical analysis

The FNA rate (FNAR) was determined by calculating the percentage of nodules recommended for FNA out of the total nodules. The unnecessary FNA rate (UFR) was computed by determining the ratio of benign nodules among the nodules that were advised to undergo FNA. The missed cancer rate (MCR) was derived by calculating the proportion of malignant nodules that were not recommended for FNA out of all malignant nodules.

Statistical analysis was conducted using SPSS 26.0 (IBM Corp., Armonk, NY, USA) and MedCalc 18.2.1 (MedCalc Software Ltd, Ostend, Belgium) software. Continuous data were presented as mean ± standard deviation (SD) and compared using the independent two-sample t-test. Categorical data were compared using the Chi-square test or Fisher’s exact test. Receiver Operating Characteristic (ROC) curves were constructed, and the Area Under ROC (AUC) was compared using the DeLong method or Z-test. Sensitivity, specificity, positive predictive value (PPV), negative predictive value (NPV), accuracy, FNAR, UFR and MCR with 95% confidence intervals (CI) were evaluated for the RSSs and compared using the McNemar or Chi-square test. A two-sided P-value of <0.05 was considered statistically significant.

## 3 Results

### 3.1 Basic characteristics

The malignant rate in the elderly group was significantly lower than that in the younger group (P =0.000). There was no statistically significant difference in gender between the two groups (P =0.119). However, the elderly group had a higher prevalence of multiple nodules and larger nodules, with a higher proportion of nodules measuring ≥20mm and a lower proportion of nodules measuring <10mm compared to the younger group (P<0.05) (**Table 1**).

**Table 1:** Basic characteristics of thyroid nodules according to age group.

Characteristics	Elderly Group	Younger Group	*t/X^2^* Value	*P*-Value
Age				
Rang (years)	60~84	18~59		
Mean (years)	65.8 ± 5.0	43.4 ± 9.2	66.3	0.000
Pathology			116.4	0.000
Benign (n, %)	570 (71.8)	1574 (50.4)		
Malignant (n, %)	224 (28.2)	1546 (49.6)		
Gender			2.4	0.119
Male (n, %)	133 (26.4)	493 (23.1)		
Female (n, %)	371 (73.6)	1641 (76.9)		
Size				
Range (mm)	2~105	2-93		
Mean (mm, means ± SD)	21.0 ± 17.3	15.8 ± 13.5	9.228	0.000
<10mm*	269 (33.9)	1442 (46.2)	54.7	0.000
10-20mm	205 (25.8)	811 (26.0)		
≥20mm*	320 (40.3)	867 (27.8)		
Single/Multiple			54.0	0.000
Single (n, %)	254 (32.0)	1450 (46.5)		
Multiple (n, %)	540 (68.0)	1670 (53.5)		

*P<0.05 between elderly group and younger group. n, number; SD, standard deviation.

### 3.2 Comparison of ultrasound features between elderly and younger groups

There were statistically significant differences in composition, echogenicity, orientation, margin and echogenic foci between the elderly group and the younger group (**Table 2**). The proportion of solid nodules was lower in the elderly group, while the mixed cystic and solid was higher than in the younger group. The proportions of hyperechoic, isoechoic and cannot classify were higher in the elderly group compared to the younger group, while the proportion of hypoechoic and markedly hypoechoic were lower than in the younger group. The taller-than-wide was less common in the elderly group than in the younger group. Smooth, ill-defined, and cannot classify margins were more common in the elderly group compared to the younger group, while irregular and extrathyroidal extension were less common in the elderly group than in the younger group. Peripheral calcifications, macrocalcification and non-calcified nodules were more common in the elderly group than in the younger group, while punctate echogenic foci were less common in the elderly group than in the younger group.

**Table 2:** Ultrasound features of thyroid nodules according to age group.

	Elderly Group (n, %)	Younger Group (n, %)	*X^2^* Value	*P*-Value
Composition			33.1	0.000
Solid or almost solid*	480 (60.5)	2206 (70.7)		
Mixed cystic and solid*	292 (36.8)	855 (27.4)		
Cystic	19 (2.4)	56 (1.8)		
Spongiform	3 (0.4)	3 (0.1)		
Echogenicity			78.5	0.000
Hyperechoic*	24 (3.0)	53 (1.7)		
Isoechoic*	323 (40.7)	898 (28.8)		
Hypoechoic*	388 (48.9)	1947 (62.4)		
Markedly hypoechoic*	24 (3.0)	151 (4.8)		
Anechoic	19 (2.4)	56 (1.8)		
Cannot classify*	16 (2.0)	15 (0.5)		
Orientation			17.7	0.000
Taller-than-wide*	124 (15.6)	700 (22.4)		
Wider-than-tall*	670 (84.4)	2420 (77.6)		
Margin			85.5	0.000
Smooth*	323 (40.7)	1005 (32.2)		
Ill-defined*	231 (29.1)	643 (20.6)		
Irregular or lobulated*	216 (27.2)	1263 (40.5)		
Extrathyroidal extension*	21 (2.6)	207 (6.6)		
Cannot classify*	3 (0.4)	2 (0.1)		
Echogenic foci^#^			83.1	0.000
Punctate echogenic foci*	168 (21.2)	1105 (35.4)		
Macrocalcification*	140 (17.6)	323 (10.4)		
Peripheral calcification*	17 (2.1)	24 (0.8)		
None*	470 (59.2)	1670 (53.5)		
Comet-tail artifacts	16 (2.0)	54 (1.7)		

*P<0.05 between elderly group and younger group.^#^ The comet-tail artifacts were recorded only in the absence of microcalcifications. Other types of calcifications could be selected simultaneously.n, number.

### 3.3 Diagnostic efficacy of suspicious ultrasound features

From **Table 3**, it was observed that the elderly group demonstrated lower sensitivity regarding hypoechoic nodules, extrathyroidal extension, and punctuate echogenic foci in comparison to the younger group (P=0.042, 0.028, 0.000, respectively). However, they showed higher sensitivity in terms of ill-defined margins compared to the younger group (P=0.035). For specificity, the elderly group demonstrated higher specificity in terms of hypoechoic compared to the younger group (P=0.036). However, they exhibited lower specificity in terms of ill-defined margins compared to the younger group (P=0.003). All ultrasound features in the **Table 3**, except for markedly hypoechoic nodules, showed lower PPV in the elderly group compared to the younger group (P<0.05). However, all ultrasound features in the **Table 3** exhibited higher NPV in the elderly group compared to the younger group (P<0.05).

**Table 3:** Diagnostic performance of partial suspicious ultrasound features.

Diagnostic Method	Groups	Sensitivity (%) (95% CI)	Specificity (%) (95% CI)	PPV (%) (95% CI)	NPV (%) (95% CI)
Solid or almost solid	Elderly	92.4 (88.9-95.9)	52.1 (48.0-56.2)	43.1* (38.7-47.6)	94.6* (92.1-97.1)
	Younger	93.1 (91.9-94.4)	51.3 (48.9-53.8)	65.3* (63.3-67.3)	88.4* (86.3-90.5)
Mixed cystic and solid	Elderly	7.6 (4.1-11.1)	51.8 (47.6-55.9)	5.8* (3.1-8.5)	58.8* (54.4-63.1)
	Younger	6.9 (5.6-8.1)	52.4 (49.9-54.9)	12.4* (10.2-14.6)	36.4* (34.4-38.4)
Hyperechoic or isoechoic	Elderly	12.9 (8.5-17.4)	44.2 (40.1-48.3)	8.4* (5.4-11.3)	56.4* (51.8-61.0)
	Younger	9.1 (7.6-10.5)	48.5 (46.0-50.9)	14.7* (12.5-17.0)	35.2* (33.2-37.2)
Hypoechoic	Elderly	76.3* (70.7-81.9)	61.9* (57.9-65.9)	44.1* (39.1-49.0)	86.9* (83.7-90.2)
	Younger	82.0* (80.1-83.9)	56.9* (54.4-59.3)	65.1* (63.0-67.2)	76.3* (73.9-78.7)
Markedly hypoechoic	Elderly	8.0 (4.4-11.6)	98.9 (98.1-99.8)	75.0 (56.3-93.7)	73.2* (70.1-76.4)
	Younger	8.6 (7.2-10.0)	98.9 (98.3-99.4)	88.1 (82.9-93.3)	52.4* (50.6-54.2)
Taller-than-wide	Elderly	32.6 (26.4-38.8)	91.1 (88.7-93.4)	58.9* (50.1-67.7)	77.5* (74.3-80.6)
	Younger	36.6 (34.2-39.0)	91.5 (90.1-92.9)	80.9* (77.9-83.8)	59.5* (57.5-61.5)
Ill-defined	Elderly	19.6* (14.4-24.9)	66.7* (62.8-70.5)	18.8* (13.8-23.8)	67.9* (64.0-71.7)
	Younger	14.2* (12.5-16.0)	73.1* (70.9-75.3)	34.2* (30.5-37.9)	46.5* (44.5-48.4)
Irregular or lobulated	Elderly	65.2 (58.9-71.5)	87.7 (85.0-90.4)	67.6* (61.3-73.9)	86.5* (83.7-89.3)
	Younger	66.6 (64.3-69.0)	85.2 (83.4-87.0)	81.6* (79.4-83.7)	72.2* (70.2-74.3)
Extrathyroidal extension	Elderly	7.1* (3.7-10.5)	99.1 (98.4-99.9)	76.2* (56.3-96.1)	73.1* (70.0-76.2)
	Younger	12.2* (10.5-13.8)	98.8 (98.3-99.3)	90.8* (86.9-94.8)	53.4* (51.6-55.2)
Punctuate echogenic foci	Elderly	41.5* (35.0-48.0)	86.8 (84.1-89.6)	55.4* (47.8-63.0)	79.1* (75.9-82.3)
	Younger	58.7* (56.2-61.1)	87.4 (85.8-89.1)	82.1* (79.8-84.3)	68.3* (66.3-70.3)

*P<0.05 between elderly group and younger group. CI, confidence interval; PPV, positive predictive value; NPV, negative predictive value.

### 3.4 Diagnostic efficacy of RSSs in elderly and younger groups

**Figure 2:**
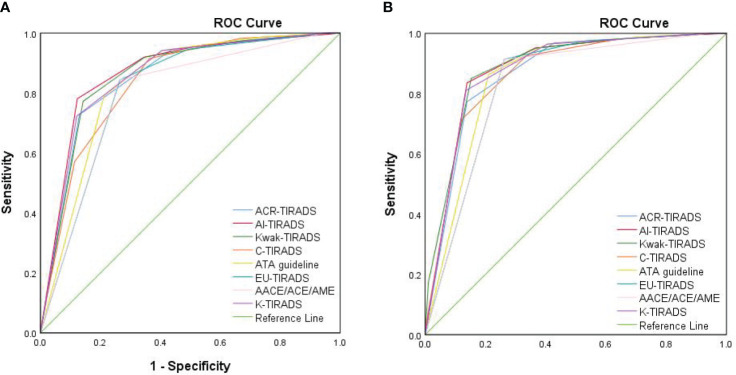
The ROC curves of eight RSSs for elderly patients and younger patients. **(A)** The ROC curve of eight RSSs for elderly patients. **(B)** The ROC curve of eight RSSs for younger patients. ROC, receiver operating characteristic curve; ACR-TIRADS, American College of Radiology Thyroid Imaging Reporting and Data System; Kwak-TIRADS, TIRADS issued by Kwak et al; C-TIRADS, Chinese-TIRADS; ATA guideline, American Thyroid Association guideline; EU-TIRADS, European TIRADS; AACE/ACE/AME, American Association of Clinical Endocrinologists, American College of Endocrinology, and Associazione Medici Endocrinology guideline; K-TIRADS, Korean TIRADS.

The AUCs for ACR, AI, Kwak, C-TIRADS, ATA, EU, AACE/ACE/AME, and K-TIRADS in the elder group were 0.854, 0.871, 0.861, 0.837, 0.832, 0.810, 0.795, and 0.859, respectively (**Figure 2A**). In the younger group, the AUCs were 0.869, 0.882, 0.887, 0.867, 0.855, 0.837, 0.828, and 0.880, respectively (**Figure 2B**). The AUCs of the elder group were consistently lower than those of the younger group in all eight RSSs (P<0.05 for Kwak, C-TIRADS and AACE/ACE/AME, P>0.05 for other five RSSs).

### 3.5 Comparison of diagnostic efficacy among different RSSs for elderly patients

The ROC showed that the cutoff value for C-TIRADS was 4B, for Kwak was 4C, for AACE/ACE/AME was 3, and for the remaining systems was 5. The highest area under the ROC curve was observed for AI, followed by Kwak, with AACE/ACE/AME exhibiting the lowest value. In terms of sensitivity, C-TIRADS demonstrated the highest level, followed by EU and AACE/ACE/AME, whereas K-TIRADS showed the lowest sensitivity. K-TIRADS displayed the highest specificity, followed by AI, while C-TIRADS exhibited the lowest specificity. The highest PPV was associated with AI, followed by K-TIRADS, while C-TIRADS presented the lowest PPV. The maximum NPV was achieved by C-TIRADS, followed by AACE/ACE/AME and EU, whereas K-TIRADS and ACR had the lowest NPV. AI showed the best accuracy, K-TIRADS came in second, and C-TIRADS exhibited the lowest accuracy (**Table 4**).

**Table 4:** Diagnostic performance of eight RSSs for elder patients.

Diagnostic Method	Sensitivity (%) (95% CI)	Specificity (%) (95% CI)	PPV (%) (95% CI)	NPV (%) (95%CI)	Accuracy (%) (95% CI)	Cut-off	AUC (95% CI)
ACR-TIRADS	72.8 (66.9-78.6)	87.0 (84.2-89.8)	68.8 (62.8-74.7)	89.0 (86.4-91.6)	83.0 (80.4-85.6)	5	0.854 (0.826-0.882)
AI-TIRADS	78.1 (72.7-83.6)	87.7 (85.0-90.4)	71.4 (65.7-77.1)	91.1 (88.7-93.5)	85.0 (82.5-87.5)	5	0.871 (0.843-0.898)
Kwak-TIRADS	77.2 (71.7-82.8)	85.8 (82.9-88.7)	68.1 (62.3-73.9)	90.6 (88.1-93.0)	83.4 (80.8-86.0)	4C	0.861 (0.833-0.889)
C-TIRADS	91.5 (87.8-95.2)	63.7 (59.7-67.6)	49.8 (44.9-54.6)	95.0 (92.8-97.2)	71.5 (68.4-74.7)	4B	0.837 (0.808-0.866)
ATA guideline	79.0 (73.6-84.4)	78.8 (75.4-82.1)	59.4 (53.8-65.0)	90.5 (87.9-93.1)	78.8 (76.0-81.7)	5	0.832 (0.803-0.861)
EU-TIRADS	84.4 (79.6,89.2)	73.5 (69.9-77.1)	55.6 (50.3-60.9)	92.3 (89.8-94.8)	76.6 (73.6-79.5)	5	0.810 (0.779-0.841)
AACE/ACE/AME	84.4 (79.6,89.2)	73.7 (70.1-77.3)	55.8 (50.4-61.1)	92.3 (89.9-94.8)	76.7 (73.8-79.6)	3	0.795 (0.761-0.828)
K-TIRADS	72.3 (66.4-78.2)	87.9 (85.2-90.6)	70.1 (64.2-76.1)	89.0 (86.4-91.6)	83.5 (80.9-86.1)	5	0.859 (0.831-0.888)

CI, confidence interval; PPV, positive predictive value; NPV, negative predictive value; AUC, area under the receiver operating characteristic curve; ACR-TIRADS, American College of Radiology Thyroid Imaging Reporting and Data System; Kwak-TIRADS, TIRADS issued by Kwak et al; C-TIRADS, Chinese-TIRADS; ATA guideline, American Thyroid Association guideline; EU-TIRADS, European TIRADS; AACE/ACE/AME, American Association of Clinical Endocrinologists, American College of Endocrinology, and Associazione Medici Endocrinology guideline; K-TIRADS, Korean TIRADS.

### 3.6 Comparison of diagnostic efficacy for elderly patients based on size thresholds

After considering the size thresholds for FNA from various guidelines, we found that the FNAR for the eight different RSSs ranged from 30.5% to 59.7%, with AI having the lowest rate and K-TIRADS the highest. The UFR ranged from 55.0% to 74.5%, with AI having the lowest rate and K-TIRADS the highest. The MCR ranged from 22.8% to 51.8%, with C-TIRADS having the lowest rate and ACR the highest (**Table 5**).

**Table 5:** Comparison of therapeutic performance for elderly patients based on size thresholds.

Guidelines	FNAR (%) (95% CI)	UFR (%) (95% CI)	MCR (%) (95% CI)
ACR-TIRADS	37.0 (33.7-40.4)	63.3 (57.7-68.8)	51.8 (45.2-58.4)
AI-TIRADS	30.5 (27.3-33.7)	55.0 (48.6-61.3)	51.3 (44.7-57.9)
Modified AI-TIRADS	33.8 (30.5-37.0)	34.3 (28.6-40.0)	21.4 (16.0-26.8)
Kwak-TIRADS	47.1 (43.6-50.6)	67.9 (63.2-72.7)	46.4 (39.8-53.0)
C-TIRADS	58.3 (54.9-61.7)	62.6 (58.2-67.1)	22.8 (17.2-28.3)
ATA guideline	57.8 (54.4-61.3)	73.6 (69.6-77.7)	46.0 (39.4-52.6)
EU-TIRADS	53.3 (49.8-56.8)	72.3 (68.1-76.6)	47.8 (41.2-54.4)
AACE/ACE/AME	51.8 (48.3-55.2)	71.8 (67.4-76.1)	48.2 (41.6-54.8)
K-TIRADS	59.7 (56.3-63.1)	74.5 (70.5-78.4)	46.0 (39.4-52.6)

FNAR, fine-needle aspiration rate; UFR, unnecessary FNA rate; MCR, missed cancer rate; CI, confidence interval; ACR-TIRADS, American College of Radiology Thyroid Imaging Reporting and Data System; Kwak-TIRADS, TIRADS issued by Kwak et al; C-TIRADS, Chinese-TIRADS; ATA guideline, American Thyroid Association guideline; EU-TIRADS, European TIRADS; AACE/ACE/AME, American Association of Clinical Endocrinologists, American College of Endocrinology, and Associazione Medici Endocrinology guideline; K-TIRADS, Korean TIRADS.

### 3.7 The modified version of AI-TIRADS with adjusted size thresholds

From the **Table 5**, we observed that after incorporating the size thresholds for FNA, all eight RSSs performed poorly. Among them, AI had the lowest FNAR and UFR, while C-TIRADS had the lowest MCR. Taken together, AI showed the best diagnostic efficacy among older adults. However, the size thresholds for FNA needed to be adjusted. Considering the specific characteristics of nodules in elderly patients, we found that by modifying the size thresholds of category 3 from ≥25 to no-FNA for all, the category 4 from ≥15 to ≥25, and the category 5 from ≥10 to ≥5, the UFR of the new modified AI TI-RADS decreased from 55.0% to 34.3%. The MCR also significantly decreased from 51.3% to 21.4%, and the FNAR only increased from 30.5% to 33.8%. The modified AI demonstrated the lowest UFR (P<0.05 compared to all eight RSSs) and MCR (P=0.733 compared to C-TIRADS and P<0.05 compared to the other seven RSSs). Additionally, it achieved nearly the lowest FNAR (P=0.172, 0.162, compared to the ACR and original AI, respectively, but P<0.05 compared to the other six RSSs).

## 4 Discussion

Among the various versions of US-based RSSs, AI-TIRADS demonstrated the best overall performance, with the largest AUC, highest PPV and accuracy, nearly the highest specificity and relatively high sensitivity and NPV, which suggested that AI-TIRADS was more suitable for elderly individuals. AI-TIRADS was a simplified version of ACR based on artificial intelligence algorithms, sharing the same risk stratification and the same thresholds for FNA with ACR, thus maintaining its excellent diagnostic efficacy and reducing UFR. Furthermore, it excluded the scoring of several ultrasound indicators, making the evaluation process simpler and thereby improving user-friendliness. Previous studies have confirmed that AI had similar or even higher diagnostic value compared to ACR (24, 25), which was consistent with our study findings.

Combining the size thresholds for FNA, we found that the FNAR for various guidelines ranged from 30.5% to 59.7%, the UFR ranged from 55.0% to 74.5%, and the MCR ranged from 22.8% to 51.8%. Despite AI demonstrating the best performance, its UFR and MCR were as high as 55.0% and 51.3%, respectively. This indicated that the current size thresholds in existing guidelines were not suitable for the elderly, including the ACR/AI, which had been reported to have the lowest UFR in previous literatures (12, 14). One possible reason was that thyroid nodules in the elderly population were generally larger and had a higher prevalence of benign nodules. The results of this study also corroborated this point. A study reported that the malignancy rate of thyroid nodules in individuals aged 20-49 was 17.1-22.9%, but it decreased to only 12.6% in those aged 70 and above (17). Hence, the size thresholds that were suitable for the general population may have been relatively low for elderly individuals, resulting in a higher rate of UFR. For elderly patients, careful consideration should have been given to surgical indications because surgery for this age group not only implied treatment but also posed a significant risk due to potential morbidity associated with surgical interventions, particularly for those frail elderly individuals (26). Advancing patient age should be a factor to consider when dealing with thyroid nodules (27). As AI demonstrated the best overall performance in the diagnostic value for elderly individuals, however, with high NPV and MCR, we adjusted the size thresholds of FNA for AI. In this study, the malignancy rate of category 3 nodules in the elderly group was only 7.6% (9/119), and among them, only 33.3% (3/9) had a size of ≥25mm. These nodules could be adequately monitored through follow-up (28). Therefore, we recommend follow-up instead of FNA for category 3 nodules. For category 4, the malignancy rate was 19.6% (31/158), and we recommended adjusting the size threshold from 15mm to 25mm. With these changes above, we reduced the number of FNA nodules by 60.7% (from 122 to 48), while also avoiding unnecessary FNA for 68.3% of benign nodules (from 101 to 32). There was only a slight increase of 5 missed diagnoses (from 19 to 24).

However, for category 5 nodules, given the high malignancy rate and the higher likelihood of aggressive cancer in elderly individuals, which accounts for almost all thyroid-related deaths (26, 29), we have lowered the size threshold for grade 5 nodules from 10mm to 5mm, thus avoiding 82.8% of cancers being missed (from 87 to 15). With all the adjustments implemented, the modified AI-TIRADS showed a significant decrease in the UFR and MCR (UFR: before vs. after adjustments: 55.0% vs. 34.3%; MCR: before vs. after adjustments: 51.3% vs. 21.4%; both P=0.000). Although the FNAR increased slightly, there was no statistically significant difference compared to the ACR and original AI (P=0.172, 0.162, respectively), and it remained lower than the other six RSSs.

In this study, the ROC analysis yielded a diagnostic threshold of 4A for C-TIRADS, which differed from the previously used 4B threshold in the general population (30). This disparity in threshold selection contributed to the higher sensitivity and lower specificity observed in this research. The possible reason was that C-TIRADS only utilized a few key suspicious US signs, including solid, markedly hypoechoic, ill-defined/irregular margin or extrathyroidal extension, vertical orientation, and microcalcifications. These features were generally less sensitive in the elderly population, especially the presence of microcalcifications. As a result, C-TIRADS tended to yield lower scores in the elderly population, leading to a lower diagnostic cutoff than in previous studies. Additionally, C-TIRADS did not account for the highly sensitive feature of hypoechoic nodules, which partly explained the superior diagnostic performance observed in Kwak (3), a similar classification approach with C-TIRADS. Moreover, C-TIRADS assigned 1 point for ill-defined margin. While in the elderly population, ill-defined margin exhibited low specificity and PPV (66.7% and 18.8%, respectively), which also contributed to the divergence between C-TIRADS and Kwak’s.

It is worth mentioning that in this study, the unclassified nodules in the ATA guidelines were grouped with intermediate-suspicion categories, which was similar to the classification method of K-TIRADS (9). However, the AUC of K-TIRADS was found to be superior to ATA in this study. Upon analyzing the data, the difference was observed in mixed cystic and solid nodules with suspicious US features. ATA categorized mixed cystic and solid nodules with suspicious US features into the high suspicious category (TR-5). In contrast, K-TIRADS classified them, along with isoechoic nodules with suspicious US features, into TR-4, with only solid hypoechoic nodules with malignant features classified into TR-5. This emphasized the predictive ability of solid hypoechoic nodules for malignancy. The data from this study also confirmed this point. In the elderly group, the PPV of solid nodules was 43.1%, whereas mixed cystic and solid nodules were only 5.8%. Hypoechoic nodules, although less correlated with malignancy in the elderly group compared to the younger group, still reached 44.1%, while hyperechoic or isoechoic nodules were only 8.4%. This indicates that in clinical practice, paramount significance should be given to the predictive ability of solid hypoechoic nodules for malignancy.

This study had several limitations. Firstly, it was conducted at a single center, which may have limited the generalizability of the findings. Multi-center studies would have been necessary to validate and strengthen the results in the future. Secondly, due to the limited number of patients aged 80 and above, the study did not compare different age groups within the elderly population. Thirdly, as this study was retrospective in nature, there might have been some limitations in image interpretation. Conducting further prospective studies would be essential to establish more definitive conclusions.

## 5 Conclusion

All eight RSSs showed acceptable diagnostic efficacy in elderly patients, albeit lower compared to younger patients. Among these RSSs, AI demonstrated the highest overall diagnostic efficacy. By adjusting the size thresholds, the AI TIRADS achieved the lowest UFR, MCR, and nearly the lowest FNAR, thus offering enhanced guidance for clinical practice.

## Data availability statement

The raw data supporting the conclusions of this article will be made available by the authors, without undue reservation.

## Ethics statement

The studies involving humans were approved by the scientific research and clinical trials ethics committee of the First Affiliated Hospital of Zhengzhou University. The studies were conducted in accordance with the local legislation and institutional requirements. The ethics committee/institutional review board waived the requirement of written informed consent for participation from the participants or the participants’ legal guardians/next of kin because this study was retrospective and only necessary clinical data were collected.

## Author contributions

XM: Data curation, Investigation, Project administration, Writing – original draft. JY: Conceptualization, Investigation, Methodology, Project administration, Writing – review & editing, Writing – original draft. YH: Conceptualization, Formal Analysis, Investigation, Supervision, Writing – review & editing. YC: Data curation, Formal Analysis, Investigation, Software, Validation, Writing – review & editing. KC: Conceptualization, Supervision, Validation, Writing – review & editing.
